# (2,2′-Bipyridine-κ^2^ *N*,*N*′)dibromido­palladium(II) dichloro­methane solvate

**DOI:** 10.1107/S1600536809039701

**Published:** 2009-10-03

**Authors:** Nam-Ho Kim, Kwang Ha

**Affiliations:** a School of Applied Chemical Engineering, The Research Institute of Catalysis, Chonnam National University, Gwangju 500-757, Republic of Korea

## Abstract

In the title compound, [PdBr_2_(C_10_H_8_N_2_)]·CH_2_Cl_2_, the Pd^2+^ ion is four-coordinated in a slightly distorted square-planar environment by two N atoms of the chelating 2,2′-bipyridine ligand and two bromide ions. The compound displays intra­molecular C—H⋯Br hydrogen bonds and pairs of complex mol­ecules are assembled by inter­molecular C—H⋯Br hydrogen bonds. These pairs are connected by additional C—H⋯Br hydrogen bonds, forming a layer structure extending parallel to (011). Inter­molecular π–π inter­actions between the pyridine rings of the ligand are also present, the shortest centroid–centroid distance being 4.090 (9) Å.

## Related literature

For the crystal structures of [Pd*X*_2_(bipy)] (*X* = Cl or Br), see: Maekawa *et al.* (1991 ▶); Smeets *et al.* (1997 ▶). For the crystal structure of [PdCl_2_(bipy)]·CH_2_Cl_2_ which is isotypic to the title compound, see: Vicente *et al.* (1997 ▶); Kim *et al.* (2009*a* ▶). For related Pt(II, IV)-bipyridine complexes, see: Osborn & Rogers (1974 ▶); Hambley (1986 ▶); Sartori *et al.* (2005 ▶); Momeni *et al.* (2007 ▶); Kim *et al.* (2009*b* ▶).
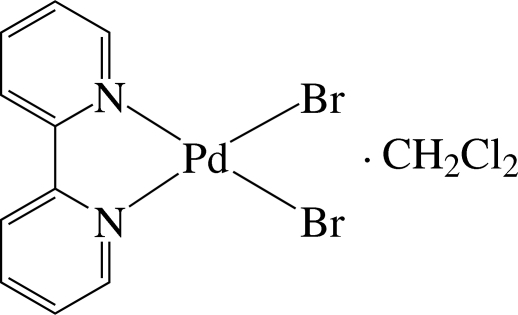


## Experimental

### 

#### Crystal data

[PdBr_2_(C_10_H_8_N_2_)]·CH_2_Cl_2_
*M* *_r_* = 507.33
Triclinic, 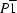

*a* = 8.9323 (10) Å
*b* = 9.3035 (10) Å
*c* = 10.0113 (11) Å
α = 72.882 (2)°
β = 67.292 (2)°
γ = 80.995 (2)°
*V* = 732.60 (14) Å^3^
*Z* = 2
Mo *K*α radiation
μ = 7.07 mm^−1^
*T* = 200 K
0.22 × 0.15 × 0.11 mm

#### Data collection

Bruker SMART 1000 CCD diffractometer
Absorption correction: multi-scan (*SADABS*; Bruker, 2000 ▶) *T*_min_ = 0.707, *T*_max_ = 1.000
5486 measured reflections
3574 independent reflections
2195 reflections with *I* > 2σ(*I*)
*R*_int_ = 0.038

#### Refinement

*R*[*F* ^2^ > 2σ(*F* ^2^)] = 0.068
*wR*(*F* ^2^) = 0.188
*S* = 1.14
3574 reflections
163 parameters
H-atom parameters constrained
Δρ_max_ = 2.13 e Å^−3^
Δρ_min_ = −3.43 e Å^−3^

### 

Data collection: *SMART* (Bruker, 2000 ▶); cell refinement: *SAINT* (Bruker, 2000 ▶); data reduction: *SAINT*; program(s) used to solve structure: *SHELXS97* (Sheldrick, 2008 ▶); program(s) used to refine structure: *SHELXL97* (Sheldrick, 2008 ▶); molecular graphics: *ORTEP-3* (Farrugia, 1997 ▶) and *PLATON* (Spek, 2009 ▶); software used to prepare material for publication: *SHELXL97*.

## Supplementary Material

Crystal structure: contains datablocks global, I. DOI: 10.1107/S1600536809039701/wm2260sup1.cif

Structure factors: contains datablocks I. DOI: 10.1107/S1600536809039701/wm2260Isup2.hkl

Additional supplementary materials:  crystallographic information; 3D view; checkCIF report

## Figures and Tables

**Table 1:** Hydrogen-bond geometry (Å, °)

*D*—H⋯*A*	*D*—H	H⋯*A*	*D*⋯*A*	*D*—H⋯*A*
C1—H1⋯Br2	0.95	2.69	3.313 (13)	124
C2—H2⋯Br2^i^	0.95	2.84	3.659 (16)	145
C10—H10⋯Br1	0.95	2.72	3.343 (14)	124
C11—H11*A*⋯Br2	0.99	2.92	3.693 (15)	135
C11—H11*B*⋯Br1^ii^	0.99	2.81	3.668 (16)	145

Symmetry codes: (i) 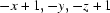; (ii) 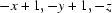.

**Fig. 1.**
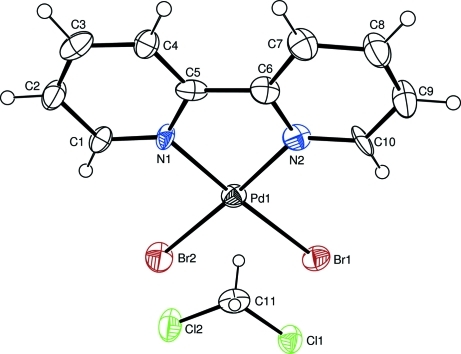
The structure of the title compound, with displacement ellipsoids drawn at the 40% probability level for non-H atoms. H atoms are displayed as spheres of arbitrary radius.

**Fig. 2.**
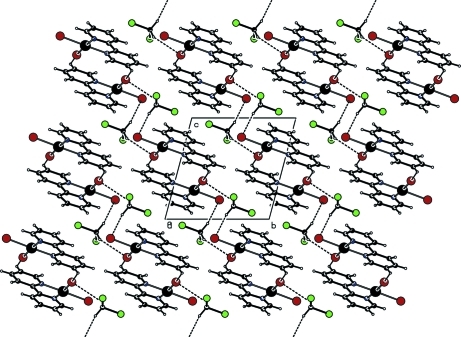
Crystal packing of the title compound. Hydrogen-bonding interactions are drawn with dashed lines. Intramolecular C—H···Br hydrogen bonds are omitted.

## supplementary crystallographic information

### Comment

The asymmetric unit of the title compound, [PdBr_2_(C_10_H_8_N_2_)].CH_2_Cl_2_, contains a neutral Pd^II^ complex and a solvent molecule (Fig. 1). The compound crystallizes in the triclinic space group *P*1, whereas the previously reported solvent-free complex [PdBr_2_(C_10_H_8_N_2_)] crystallizes in the monoclinic space group *C*2/*c* (Smeets *et al.*, 1997).

In the title complex, the Pd^2+^ ion is four-coordinated in a slightly distorted square-planar environment by two N atoms of the 2,2'-bipyridine (bipy) ligand and two Br anions. The main contribution to the distortion is the tight N1—Pd1—N2 chelate angle (80.6 (5)°), which results in a non-linear *trans* arrangement (<N1—Pd1—Br1 = 175.7 (3)° and <N2—Pd1—Br2 = 175.7 (4)°). Each of the two Pd1—N and Pd1—Br bond lengths are almost equal, (Pd1—N: 2.042 (9) and 2.051 (11) Å; Pd1—Br 2.4182 (18) and 2.4044 (19) Å), and close to those reported for [PdBr_2_(C_10_H_8_N_2_)] (Smeets *et al.*, 1997). The compound displays inter- and intramolecular C—H···Br hydrogen bonds (Table 1). Pairs of complex molecules are assembled by intermolecular hydrogen bonds, and the dichloromethane solvent molecules connect the pairs by intermolecular hydrogen bonds, thereby forming a layer structure extending parallel to (011) (Fig. 2). There may also be intermolecular π-π interactions between adjacent pyridine rings of the lignad (the symmetry operation for second plane is -*x*,-*y*,-*z*), with a shortest centroid-centroid distance of 4.090 (9) Å, and the planes are parallel and shifted for 1.758 Å.

For the crystal structures of related palladium(II) halogenides with bipyridine ligands, [Pd*X*_2_(bipy)], where *X* = Cl or Br, see: Maekawa *et al.* (1991); Smeets *et al.* (1997). For [PdCl_2_(bipy)].CH_2_Cl_2_ that crystallizes isotypically with the title compound, see: Vicente *et al.* (1997); Kim *et al.* (2009*a*). For related Pt(II, IV)-bipyridine complexes, see: Osborn & Rogers (1974); Hambley (1986); Sartori *et al.* (2005); Momeni *et al.* (2007); Kim *et al.* (2009*b*).

### Experimental

To a solution of K_2_PdBr_4_ (0.100 g, 0.198 mmol) in EtOH (10 ml) was added 2,2'-bipyridine (0.031 g, 0.198 mmol), and refluxed for 4 h. The precipitate obtained was separated by filtration and washed with EtOH and water and dried under vacuum to give an orange powder (0.054 g). Crystals suitable for X-ray analysis were obtained by slow evaporation from a CH_2_Cl_2_ solution.

### Refinement

H atoms were positioned geometrically and allowed to ride on their respective parent atoms [C—H = 0.95 (CH) or 0.99 (CH_2_) Å and *U*_iso_(H) = 1.2*U*_eq_(C)]. The highest peak and the deepest hole in the final Fourier map are 1.30 Å from atom Pd1 and 0.81 Å from the same atom.

### Crystal data

**Table:**

[PdBr_2_(C_10_H_8_N_2_)]·CH_2_Cl_2_	*Z* = 2
*M**_r_* = 507.33	*F*(000) = 480
Triclinic, *P*1	*D*_x_ = 2.300 Mg m^−^^3^
Hall symbol: -P 1	Mo *K*α radiation, λ = 0.71073 Å
*a* = 8.9323 (10) Å	Cell parameters from 1623 reflections
*b* = 9.3035 (10) Å	θ = 2.3–26.7°
*c* = 10.0113 (11) Å	µ = 7.07 mm^−^^1^
α = 72.882 (2)°	*T* = 200 K
β = 67.292 (2)°	Block, dark orange
γ = 80.995 (2)°	0.22 × 0.15 × 0.11 mm
*V* = 732.60 (14) Å^3^	

### Data collection

**Table:**

Bruker SMART 1000 CCD diffractometer	3574 independent reflections
Radiation source: fine-focus sealed tube	2195 reflections with *I* > 2σ(*I*)
graphite	*R*_int_ = 0.038
φ and ω scans	θ_max_ = 28.3°, θ_min_ = 2.3°
Absorption correction: multi-scan (*SADABS*; Bruker, 2000)	*h* = −11→11
*T*_min_ = 0.707, *T*_max_ = 1.000	*k* = −12→12
5486 measured reflections	*l* = −13→10

### Refinement

**Table:**

Refinement on *F*^2^	Primary atom site location: structure-invariant direct methods
Least-squares matrix: full	Secondary atom site location: difference Fourier map
*R*[*F*^2^ > 2σ(*F*^2^)] = 0.068	Hydrogen site location: inferred from neighbouring sites
*wR*(*F*^2^) = 0.188	H-atom parameters constrained
*S* = 1.14	*w* = 1/[σ^2^(*F*_o_^2^) + 21.2252*P*] where *P* = (*F*_o_^2^ + 2*F*_c_^2^)/3
3574 reflections	(Δ/σ)_max_ < 0.001
163 parameters	Δρ_max_ = 2.13 e Å^−^^3^
0 restraints	Δρ_min_ = −3.43 e Å^−^^3^

### Special details

**Table:**

Geometry. All e.s.d.'s (except the e.s.d. in the dihedral angle between two l.s. planes) are estimated using the full covariance matrix. The cell e.s.d.'s are taken into account individually in the estimation of e.s.d.'s in distances, angles and torsion angles; correlations between e.s.d.'s in cell parameters are only used when they are defined by crystal symmetry. An approximate (isotropic) treatment of cell e.s.d.'s is used for estimating e.s.d.'s involving l.s. planes.
Refinement. Refinement of *F*^2^ against ALL reflections. The weighted *R*-factor *wR* and goodness of fit *S* are based on *F*^2^, conventional *R*-factors *R* are based on *F*, with *F* set to zero for negative *F*^2^. The threshold expression of *F*^2^ > σ(*F*^2^) is used only for calculating *R*-factors(gt) *etc*. and is not relevant to the choice of reflections for refinement. *R*-factors based on *F*^2^ are statistically about twice as large as those based on *F*, and *R*- factors based on ALL data will be even larger.

### Fractional atomic coordinates and isotropic or equivalent isotropic displacement parameters (Å^2^)

**Table:**

	*x*	*y*	*z*	*U*_iso_*/*U*_eq_	
Pd1	0.17533 (13)	0.20690 (12)	0.28502 (12)	0.0321 (3)	
Br1	0.1261 (2)	0.47367 (16)	0.19026 (18)	0.0424 (4)	
Br2	0.3633 (2)	0.26600 (18)	0.3779 (2)	0.0491 (4)	
N1	0.2025 (13)	−0.0210 (11)	0.3616 (12)	0.028 (2)	
N2	0.0139 (13)	0.1409 (13)	0.2168 (14)	0.036 (3)	
C1	0.3048 (18)	−0.0927 (14)	0.4310 (15)	0.036 (3)	
H1	0.3742	−0.0363	0.4462	0.043*	
C2	0.3107 (19)	−0.2513 (16)	0.4819 (17)	0.042 (4)	
H2	0.3822	−0.3021	0.5325	0.051*	
C3	0.2123 (18)	−0.3299 (16)	0.4572 (17)	0.043 (4)	
H3	0.2171	−0.4369	0.4870	0.052*	
C4	0.1059 (19)	−0.2536 (15)	0.3888 (16)	0.039 (3)	
H4	0.0338	−0.3090	0.3760	0.047*	
C5	0.1007 (15)	−0.0985 (15)	0.3378 (15)	0.033 (3)	
C6	−0.0030 (16)	−0.0117 (16)	0.2607 (16)	0.036 (3)	
C7	−0.1125 (18)	−0.0734 (18)	0.2308 (17)	0.043 (4)	
H7	−0.1228	−0.1793	0.2599	0.052*	
C8	−0.210 (2)	0.0237 (19)	0.1559 (18)	0.050 (4)	
H8	−0.2888	−0.0153	0.1361	0.060*	
C9	−0.188 (2)	0.1730 (18)	0.1129 (18)	0.049 (4)	
H9	−0.2510	0.2396	0.0603	0.059*	
C10	−0.0763 (18)	0.2292 (19)	0.1449 (17)	0.043 (4)	
H10	−0.0636	0.3348	0.1143	0.051*	
C11	0.5407 (19)	0.6259 (17)	0.1286 (19)	0.048 (4)	
H11A	0.4406	0.5743	0.2001	0.058*	
H11B	0.5875	0.5785	0.0437	0.058*	
Cl1	0.4918 (5)	0.8180 (5)	0.0621 (5)	0.0515 (10)	
Cl2	0.6808 (6)	0.6039 (5)	0.2181 (6)	0.0662 (13)	

### Atomic displacement parameters (Å^2^)

**Table:**

	*U*^11^	*U*^22^	*U*^33^	*U*^12^	*U*^13^	*U*^23^
Pd1	0.0322 (6)	0.0287 (5)	0.0372 (6)	−0.0048 (4)	−0.0124 (5)	−0.0096 (4)
Br1	0.0503 (9)	0.0281 (7)	0.0507 (10)	−0.0036 (6)	−0.0195 (7)	−0.0104 (6)
Br2	0.0533 (10)	0.0378 (8)	0.0684 (12)	−0.0122 (7)	−0.0345 (9)	−0.0089 (8)
N1	0.032 (6)	0.015 (5)	0.033 (6)	−0.001 (4)	−0.017 (5)	0.004 (4)
N2	0.022 (6)	0.040 (7)	0.053 (8)	−0.006 (5)	−0.013 (5)	−0.018 (6)
C1	0.051 (9)	0.015 (6)	0.037 (8)	−0.003 (6)	−0.018 (7)	0.004 (5)
C2	0.047 (9)	0.027 (7)	0.052 (10)	0.004 (6)	−0.024 (8)	−0.003 (6)
C3	0.045 (9)	0.024 (7)	0.042 (9)	−0.001 (6)	0.002 (7)	−0.005 (6)
C4	0.055 (9)	0.024 (7)	0.036 (8)	−0.003 (6)	−0.021 (7)	0.001 (6)
C5	0.020 (6)	0.041 (8)	0.040 (8)	−0.006 (6)	−0.001 (5)	−0.024 (6)
C6	0.027 (7)	0.040 (8)	0.041 (8)	−0.010 (6)	−0.009 (6)	−0.011 (6)
C7	0.041 (9)	0.046 (9)	0.043 (9)	−0.011 (7)	−0.016 (7)	−0.006 (7)
C8	0.053 (10)	0.053 (10)	0.055 (11)	−0.008 (8)	−0.035 (9)	−0.007 (8)
C9	0.051 (10)	0.048 (9)	0.054 (10)	−0.010 (8)	−0.034 (8)	0.006 (8)
C10	0.045 (9)	0.059 (10)	0.045 (9)	−0.007 (7)	−0.037 (7)	−0.014 (7)
C11	0.041 (9)	0.045 (9)	0.055 (10)	−0.016 (7)	−0.004 (8)	−0.018 (8)
Cl1	0.055 (2)	0.045 (2)	0.055 (3)	−0.0045 (18)	−0.026 (2)	−0.0050 (18)
Cl2	0.069 (3)	0.052 (3)	0.091 (4)	0.003 (2)	−0.052 (3)	−0.008 (2)

### Geometric parameters (Å, °)

**Table:**

Pd1—N1	2.042 (9)	C4—H4	0.9500
Pd1—N2	2.051 (11)	C5—C6	1.43 (2)
Pd1—Br2	2.4044 (19)	C6—C7	1.371 (19)
Pd1—Br1	2.4182 (18)	C7—C8	1.41 (2)
N1—C1	1.338 (17)	C7—H7	0.9500
N1—C5	1.370 (15)	C8—C9	1.35 (2)
N2—C10	1.317 (18)	C8—H8	0.9500
N2—C6	1.371 (17)	C9—C10	1.373 (19)
C1—C2	1.412 (17)	C9—H9	0.9500
C1—H1	0.9500	C10—H10	0.9500
C2—C3	1.36 (2)	C11—Cl2	1.757 (17)
C2—H2	0.9500	C11—Cl1	1.763 (16)
C3—C4	1.37 (2)	C11—H11A	0.9900
C3—H3	0.9500	C11—H11B	0.9900
C4—C5	1.382 (18)		
			
N1—Pd1—N2	80.6 (5)	N1—C5—C4	117.9 (13)
N1—Pd1—Br2	95.3 (3)	N1—C5—C6	117.2 (12)
N2—Pd1—Br2	175.7 (4)	C4—C5—C6	124.9 (12)
N1—Pd1—Br1	175.7 (3)	C7—C6—N2	120.9 (14)
N2—Pd1—Br1	95.1 (3)	C7—C6—C5	123.7 (13)
Br2—Pd1—Br1	88.90 (6)	N2—C6—C5	115.4 (12)
C1—N1—C5	121.3 (11)	C6—C7—C8	118.5 (15)
C1—N1—Pd1	125.6 (9)	C6—C7—H7	120.7
C5—N1—Pd1	113.0 (9)	C8—C7—H7	120.7
C10—N2—C6	119.5 (12)	C9—C8—C7	118.9 (15)
C10—N2—Pd1	126.8 (10)	C9—C8—H8	120.6
C6—N2—Pd1	113.6 (10)	C7—C8—H8	120.6
N1—C1—C2	120.6 (13)	C8—C9—C10	120.3 (15)
N1—C1—H1	119.7	C8—C9—H9	119.8
C2—C1—H1	119.7	C10—C9—H9	119.8
C3—C2—C1	118.8 (14)	N2—C10—C9	121.9 (15)
C3—C2—H2	120.6	N2—C10—H10	119.1
C1—C2—H2	120.6	C9—C10—H10	119.1
C2—C3—C4	119.4 (13)	Cl2—C11—Cl1	110.9 (8)
C2—C3—H3	120.3	Cl2—C11—H11A	109.5
C4—C3—H3	120.3	Cl1—C11—H11A	109.5
C3—C4—C5	121.9 (14)	Cl2—C11—H11B	109.5
C3—C4—H4	119.1	Cl1—C11—H11B	109.5
C5—C4—H4	119.1	H11A—C11—H11B	108.0
			
N2—Pd1—N1—C1	178.3 (12)	C3—C4—C5—N1	−2(2)
Br2—Pd1—N1—C1	−3.1 (11)	C3—C4—C5—C6	177.7 (14)
N2—Pd1—N1—C5	−2.9 (9)	C10—N2—C6—C7	0(2)
Br2—Pd1—N1—C5	175.6 (8)	Pd1—N2—C6—C7	176.6 (11)
N1—Pd1—N2—C10	179.9 (13)	C10—N2—C6—C5	179.8 (13)
Br1—Pd1—N2—C10	0.6 (13)	Pd1—N2—C6—C5	−3.5 (15)
N1—Pd1—N2—C6	3.5 (9)	N1—C5—C6—C7	−179.1 (13)
Br1—Pd1—N2—C6	−175.8 (9)	C4—C5—C6—C7	1(2)
C5—N1—C1—C2	0(2)	N1—C5—C6—N2	1.1 (18)
Pd1—N1—C1—C2	178.7 (10)	C4—C5—C6—N2	−178.6 (13)
N1—C1—C2—C3	1(2)	N2—C6—C7—C8	−1(2)
C1—C2—C3—C4	−2(2)	C5—C6—C7—C8	179.1 (14)
C2—C3—C4—C5	3(2)	C6—C7—C8—C9	2(2)
C1—N1—C5—C4	0.4 (19)	C7—C8—C9—C10	−2(3)
Pd1—N1—C5—C4	−178.4 (10)	C6—N2—C10—C9	0(2)
C1—N1—C5—C6	−179.2 (12)	Pd1—N2—C10—C9	−175.8 (12)
Pd1—N1—C5—C6	2.0 (15)	C8—C9—C10—N2	0(3)

### Hydrogen-bond geometry (Å, °)

**Table:**

*D*—H···*A*	*D*—H	H···*A*	*D*···*A*	*D*—H···*A*
C1—H1···Br2	0.95	2.69	3.313 (13)	124
C2—H2···Br2^i^	0.95	2.84	3.659 (16)	145
C10—H10···Br1	0.95	2.72	3.343 (14)	124
C11—H11A···Br2	0.99	2.92	3.693 (15)	135
C11—H11B···Br1^ii^	0.99	2.81	3.668 (16)	145

Symmetry codes: (i) −*x*+1, −*y*, −*z*+1; (ii) −*x*+1, −*y*+1, −*z*.
